# Decrease of CD4-lymphocytes and apoptosis of CD14-monocytes are characteristic alterations in sepsis caused by ventilator-associated pneumonia: results from an observational study

**DOI:** 10.1186/cc8148

**Published:** 2009-11-02

**Authors:** Aimilia Pelekanou, Iraklis Tsangaris, Antigoni Kotsaki, Vassiliki Karagianni, Helen Giamarellou, Apostolos Armaganidis, Evangelos J Giamarellos-Bourboulis

**Affiliations:** 1 4th Department of Internal Medicine, ATTIKON University Hospital, 1 Rimini Str., Athens 124 62, Greece; 2 2nd Department of Critical Care, ATTIKON University Hospital, 1 Rimini Str., Athens 124 62, Greece

## Abstract

**Introduction:**

The present study aimed to investigate changes of the immune response between sepsis due to ventilator-associated pneumonia (VAP) and sepsis due to other types of infections.

**Methods:**

Peripheral venous blood was sampled from 68 patients with sepsis within 24 hours of diagnosis; 36 suffered from VAP; 32 from other nosocomial infections, all well-matched for severity, age and sex. Blood monocytes were isolated and cultured with/without purified endotoxin (lipopolysaccharide (LPS)). Estimation of tumour necrosis factor alpha (TNFα) and interleukin-6 (IL-6) in cultures' supernatants was done by an enzyme immunoassay. Flow cytometry was used to determine subpopulations of mononuclear cells and apoptosis. To mimic pathogenesis of VAP, mononuclear cells of healthy volunteers were progressively stimulated with increased inocula of pathogens; apoptosis was determined.

**Results:**

In patients with VAP, the absolute number of CD3(+)/CD4(+) lymphocytes was significantly lower (*P *= 0.034) and apoptosis of isolated monocytes was increased (*P *= 0.007) compared to other infections. TNFα and IL-6 production from LPS-stimulated monocytes was lower in patients with VAP-related sepsis than with sepsis due to other infections. Apoptosis of monocytes was induced after in vitro stimulation of mononuclear cells by a mechanism mimicking VAP.

**Conclusions:**

Decrease of CD4-lymphocytes and immunoparalysis of monocytes are characteristic alterations of sepsis arising in the field of VAP.

## Introduction

Sepsis is an important cause of admission and mortality in intensive care units (ICU). In Europe, the Sepsis Occurrence in Acutely Ill Patients study disclosed an ICU mortality rate from sepsis ranging between 27% and 54% depending on the severity [1]. In the USA, 215,000 deaths are reported annually due to sepsis [2].

Ventilator associated pneumonia (VAP) is the most common nosocomial infection and the leading cause of sepsis in the ICU. Up to 28% of patients receiving mechanical ventilation will eventually develop VAP, with a mortality rate of up to 70% [3-7].

Various explanations have been proposed for the increased mortality of patients with VAP. One previous study from our group in a cohort of 90 patients with sepsis and VAP mainly caused by Gram-negative bacteria disclosed an association between derangements of the innate immune system and mortality. More precisely, patients with early monocyte apoptosis greater than 50% were less likely to die compared with those exhibiting monocyte apoptosis lower than 50% [8]. However, it was not studied whether apoptosis of monocytes is the only detrimental alteration of the immune response linked to final outcome or if other changes of the adaptive immune system may have an effect as well. It should also be noted that this latter study was focused on patients with sepsis due to VAP, whereas sepsis of other infectious etiologies may differ in terms of its immune responses.

The present study was designed to unravel the unique features of the innate and adaptive immune responses of patients with sepsis due to VAP compared with patients with sepsis due to other infectious diseases and to propose a mechanism mediating these differences.

## Materials and methods

### Study population

A total of 68 patients were enrolled in the study. Patients were hospitalized in the second Department of Critical Care Medicine and in the fourth Department of Internal Medicine of ATTIKON University Hospital in Athens. The study was approved by the Ethics Committee of the hospital. Written informed consent was provided by patients or their relatives. All patients were older than 18 years. Exclusion criteria included neutropenia (≤500 neutrophils/μl), HIV infection or oral intake of corticosteroids at a dose equal to or higher than 1 mg/kg equivalent prednisone for at least one month.

All sequential admissions with sepsis, severe sepsis or septic shock were screened for enrolment during the period January 2006 to June 2007. Patients finally enrolled were those with septic syndrome due to VAP and those with septic syndrome caused by other types of infection, namely acute pyelonephritis, primary bacteremia, intraabdominal infection, community-acquired pneumonia (CAP) and hospital-acquired pneumonia (HAP), provided that they were well-matched to patients with VAP by age, sex, underlying conditions and disease severity.

Sepsis was defined as any microbiologically documented or clinically diagnosed infection accompanied by at least two of the following: core temperature above 38°C or below 36°C; pulse rate above 90 beats/minute; respiratory rate above 20 breaths/minute or partial pressure of carbon dioxide (pCO2) below 32 mmHg; and leukocytosis (white blood cells (WBC) >12,000 cells/μl) or leukopenia (WBC <4000 cels/μl) or presence of immature forms above 10% of total WBC count [9,10].

Severe sepsis was defined as sepsis aggravated by the acute dysfunction of at least one organ. Acute organ dysfunction was defined as follows: acute respiratory distress syndrome, as any value of partial oxygen pressure/fraction of inspired oxygen (pO2/FiO2) less than 200 and diffuse bilateral infiltrations in chest X-ray; acute renal failure, as the production of less than 0.5 ml urine/kg/hour for at least two hours, provided that the negative fluid balance of the patient was corrected; metabolic acidosis, as any pH below 7.30 or any base deficit above 5 mEq/l and serum lactate at least more than twice the upper normal value; and acute coagulopathy, as any platelet count below 100,000 cells/μl or International Normalized Ratio above 1.5 [9,10].

Septic shock was defined as sepsis accompanied by systolic arterial pressure lower than 90 mmHg necessitating the administration of inotropic agents [9,10].

Diagnosis of VAP was established if all the following criteria were met: intubation and mechanical ventilation for at least 48 hours prior to diagnosis; a new or progressive infiltrate on a chest X-ray; purulent tracheobronchial secretions; and Clinical Pulmonary Infection Score (CPIS) more than six [11-14].

Acute pyelonephritis was diagnosed in any patient presenting with all the following: fever, lumbar tenderness or radiological findings consistent with acute pyelonephritis, and pyuria defined as more than 10 WBCs/high power field or positive (+3) dipstick of urine for leukocyte esterase [15].

A diagnosis of intraabdominal infection was made in patients with temperature above 38°C or below 36°C, leukocytosis (WBC >12,000 cells/μl) and radiological findings consistent with an intraabdominal infection [15].

Primary bacteremia was defined as any positive blood culture for Gram-positive or Gram-negative microorganisms in the absence of any well-defined focus of infection, including intravascular-access devices [15].

Criteria required for the diagnosis of CAP and HAP included the presence of a new infiltrate on a chest X-ray along with two of the following: fever, leukocytosis or leukopenia, and/or purulent sputum. Pneumonia was considered as: CAP whenever the patient did not report any past hospitalization for the past 90 days or stay in a long-term care facility; or HAP when presenting more than 48 hours after hospital admission in any patient not requiring mechanical ventilation [14-16].

Patients were followed up for 28 days. A complete diagnostic work-up was performed comprising history, clinical examination, blood cell counts and biochemistry, blood cultures, chest X-ray, and chest and/or abdominal computed tomography scans if considered necessary. Quantitative cultures of urine or tracheobronchial secretions (TBS) were performed and interpreted as previously described [17] depending on the patient's underlying infection. Within the first 24 hours of the advent of signs of sepsis, 15 ml of heparinized peripheral venous blood was sampled after puncture of one forearm vein under sterile conditions.

### Laboratory techniques

For the flow cytometric analysis, red blood cells were lysed with ammonium chloride 1 mM and WBCs were washed three times with PBS (pH 7.2; Merck, Darmstadt, Germany). WBCs were then stained with fluorocolour-conjugated monoclonal antibodies against CD3, CD4, CD8, CD(16+56), CD19 and with the protein annexin-V and propidium iodine (PI) (Immunotech, Marseille, France), and incubated for 15 minutes in the dark. Fluorocolours used were fluorescein isothiocyanate (FITC; emission 525 nm; Immunotech, Marseille, France), phycoerythrin (PE; emission 575 nm; Immunotech, Marseille, France), ECD (emission 613 nm, Immunotech, Marseille, France) and PC5 (emission 670 nm, Immunotech, Marseille, France). The following combinations were applied: anti-CD3(FITC)/CD4(PE), anti-CD3(FITC)/CD8(PE), anti-CD3(FITC)/CD(16+56)(PE), anti-CD19(FITC), annexin-V(FITC)/CD4(PE)/PI (PC5), and annexin-V(FITC)/anti-CD8(PE)/PI(PC5). Cells that stained positive for annexin-V and negative for PI were considered apoptotic.

Flow-cytometric analysis was performed on an EPICS XL/MSL flow cytometer (Beckman Coulter Co, Miami, FL, USA) with gating for mononuclears based on their characteristic forward and side scattering.

For the isolation of monocytes, blood was layered over Ficoll Hypaque (Biochrom, Berlin, Germany) and centrifuged. Isolated peripheral blood mononuclear cells (PBMCs) were washed three times with PBS (pH 7.2) and incubated with RPMI 1640 media enriched with 10% fetal bovine serum (FBS) and 2 mM glutamine, 100 U/ml penicillin G and 0.1 mg/ml streptomycin (Sigma Co, St Louis, MO, USA) in 75 cm^3 ^flasks. After one hour of incubation at 37°C in 5% CO2, non-adherent cells were removed. Adherent monocytes were thoroughly washed with Hank's solution (Biochrom, Berlin, Germany), harvested with a 0.25% trypsin/0.02% ethylenediamine tetraacetic acid (EDTA) solution (Biochrom, Berlin, Germany). Their purity was more than 95% as defined after staining with anti-CD14 and analysis by a flow cytometer.

Isolated monocytes were counted in a Neubauer plate by trypan blue exclusion of dead cells, distributed in two wells of a 12-well plate and cultured with RPMI 1640 media supplemented with 10% FBS and 2 mM glutamine with or without the addition of 10 ng/ml of purified endotoxin (lipopolysaccharide (LPS)) derived from *Escherichia coli *O155:H5 (Sigma Co, St Louis, MO, USA). After incubation for 24 hours at 37°C in a 5% CO2 atmosphere, supernatants were collected and stored at -70°C until assayed for cytokines.

Estimation of TNFα and IL-6 in supernatants was performed by an ELISA (Diaclone, Paris, France). Lowest detection limits were 15.75 pg/ml for TNFα and 6.25 pg/ml for IL-6. Concentrations were adjusted as pg/10^4 ^live cells.

In an attempt to explain our findings, PBMCs of healthy volunteers were exposed to isolates of TBS from patients with VAP and to blood isolates of patients with bloodstream infections enrolled in this study. Current theories attribute pathogenesis of VAP to the aspiration of microbes colonizing the oropharynx in the lower respiratory tract. According to the theories, bacteria replicate gradually and when their growth surpasses a certain threshold then VAP develops [18,19]. In an attempt to reproduce the above sequence of events *in vitro*, PBMCs were isolated from five healthy volunteers as described above. They were distributed in wells of a 12-well plate in RPMI 1640 media supplemented with 10% FBS and 2 mM glutamine, 100 U/ml penicillin G and 0.1 mg/ml streptomycin (Sigma Co, St Louis, MO, USA). These PBMCs were stimulated by four different isolates: one of *Acinetobacter baumannii *and another of *Pseudomonas aeruginosa *isolated at a count of 1 × 10^6 ^cfu/ml or more from TBS of two different patients with VAP; and one of *A. baumannii *and another of *P. aeruginosa *isolated from blood of two different patients with bacteremia. All isolates were grown for 12 hours in Mueller-Hinton broth (Oxoid Ltd, London, UK) in a shaking-water bath at 37°C. Then a log-phase inoculum of 5 × 10^7 ^cfu/ml was prepared in Mueller-Hinton broth using the 0.5 standard of the McFarland climax. Appropriate amounts of that inoculum were used for cell stimulation in four different patterns, as follows.

Pattern A was non-stimulated PBMCs incubated for 4.75 hours in growth medium at 37°C in 5% CO2.

Pattern B was sequential stimulation in three steps mimicking pathogenesis of VAP. In the first step, PBMCs were exposed for 15 minutes at 37°C in 5% CO2 in 1 × 10^3 ^cfu/ml of each of the VAP pathogens. Then the plate was centrifuged, supernatants were discarded and the cell pellet was dissolved in 2.4 ml of growth medium. In the second step, the same procedure as in the first step was repeated after two hours. In the third step, after two hours of incubation at 37°C in a 5% CO2 atmosphere, PBMCs were stimulated with 1 × 10^6 ^cfu/ml of each of the two pathogens for 30 minutes. These inocula were selected for stimulation in an attempt to generate conditions of bacterial growth similar to those existing in patients with VAP. Then, the plate was centrifuged.

Pattern C was an abrupt stimulation with VAP pathogens. The first two steps of pattern B were performed but instead of stimulation with 1 × 10^3 ^cfu/ml inoculum, Mueller-Hinton broth was added in the plates. The third step was repeated as in pattern B.

Pattern D was an abrupt stimulation with pathogens causing bacteremia mimicking the pathogenesis of bacteremia. After incubation for 4.15 hours at 37°C in 5% CO_2 _PBMCs were exposed for 30 minutes to 1 × 10^6 ^cfu/ml of each of the two pathogens causing bacteremia. Then the plate was centrifuged.

For all the above patterns, after centrifugation of the plate and removal of supernatants, adherent cells were harvested with a 0.25% trypsin/0.02% EDTA solution (Biochrom, Berlin, Germany). Flow cytometric analysis of apoptosis was performed after staining collected cells with annexin-V(FITC)/anti-CD4(PE)/PI(PC5) and annexin-V(FITC)/anti-CD14(PE)/PI(PC5). To exclude debris or red blood cells, collected cells were also stained with anti-CD45 (ECD); their purity was more than 95%.

### Statistical analysis

Septic patients were divided in two groups, those with VAP and those suffering from other infections. Results were expressed as means (standard deviation) for parametric variables and as medians (interquartile range) for non-parametric variables. Comparisons of baseline quantitative characteristics between groups were performed by the Student's t-test and of baseline qualitative characteristics by the chi-squared test. Comparisons of non-parametric quantitative characteristics between groups were performed by the Mann-Whitney U test.

Both groups of patients were additionally divided in two subgroups each, depending on the positive response of monocytes to LPS-stimulation with or without TNFα production. A more than five-fold increase of TNFα production following stimulation was considered a positive response. Survival of two subgroups was estimated by Kaplan-Meier analysis; comparisons were performed by the log-rank test.

Apoptosis of each pattern of stimulation of PBMCs was expressed by means (standard error); comparisons were performed by analysis of variance after Bonferroni correction. Any value of *P *below 0.05 was considered significant.

## Results

Clinical characteristics of patients enrolled in the study are presented in Table 1. Other infections included pyelonephritis (7 patients), primary bacteremia (10 patients), intraabdominal infection (12 patients), CAP (1 patient) and HAP (2 patients). No differences were found between patients with VAP and patients with other infections regarding sex, age, disease severity (Acute Pathophysiology and Chronic Health Evaluation II score), WBC absolute count and differentiate, as well as the use of corticosteroids for the treatment of septic syndrome. More frequent co-morbidities were chronic obstructive pulmonary disease, diabetes mellitus, congestive heart failure and chronic renal failure, but no difference between groups was observed. Among patients who developed VAP only two had initially presented with other infections, namely peritonitis and cholecystitis, and among patients with other infections only one was primarily hospitalized because of an intraabdominal abscess.

**Table 1:** Clinical characteristics of patients with sepsis due to VAP (n = 36) and sepsis caused by other infections (n = 32).

	VAP	Other infections	*P*
Male/female	23/13	14/18	0.099
Age (years)	68.88 (15.57)	64.41 (19.76)	0.300
Sepsis/severe sepsis/septic shock	7/22/7	15/7/10	0.411
APACHE II score	18.25 (4.31)	15.33 (5.03)	0.200
WBCs (/μl)	11530 (1179)	14530 (1469)	0.141
Monocytes	234 (149)	311 (49)	0.422
Lymphocytes	629 (896)	685 (172)	0.922
Neutrophils	8299 (1342)	9180 (1371)	0.642
Use of steroids (n, %)	12 (33.33)	13 (28.12)	0.501
Comorbidities (n, %)			
COPD	9 (25.00)	3 (9.38)	0.083
DM	9 (25.00)	6 (18.75)	0.439
CHF	3 (8.33)	7 (21.87)	0.206
CRF	4 (11.11)	2 (6.25)	0.414
Prior infections (n)	2	1	
Number of failing organs (n)			
Two or more	8	11	
Duration of hospitalization (days)	18.93 (20.84)	19.04 (27.90)	0.151
Duration of mechanical ventilation (days)	18.34 (21.11)	26.76 (29.1)	0.525
Bacterial causes (n, %)			
*P. aeruginosa*	12 (33.33)	4 (12.50)	
*A. baumannii*	7 (19.44)	5 (15.62)	
*K. pneumoniae*	3 (8.33)	5 (15.62)	
*E. coli*	1 (2.77)	5 (15.62)	
*Ent. cloacae*	2 (5.55)	2 (6.25)	

Values are expressed as means (standard deviation).

APACHE = Acute Physiology and Chronic Health Evaluation; CHF = congestive heart failure; CRF = chronic renal failure; COPD = chronic pulmonary obstructive disease; DM = diabetes mellitus; VAP = ventilator-associated pneumonia; WBCs = white blood cells.

Flow-cytometric data of septic patients with VAP compared to those with other infections are shown in Table 2. The absolute number of CD3(+)/CD4(+) cells was significantly lower in patients with VAP than with other infections (*P *= 0.034). Apoptosis of isolated monocytes was increased in VAP compared with other infections (*P *= 0.007).

**Table 2:** Flow-cytometric data of patients with sepsis due to VAP and sepsis caused by other nosocomial infections.

	VAP	Other infections	*P*
CD3(+)/CD4(+)	208.52 (192)	280.68 (508.7)	0.034
CD3(+)/CD8(+)	114.8 (123.26)	102.49 (244.7)	0.787
CD3(+)/CD(16+56)(+)	18.09 (34.41)	15.11 (49.49)	0.940
*Natural killer cells	26.98 (62.94)	39.61 (45.22)	0.463
CD19(+)	28.00 (70.00)	37.93 (33.82)	0.219
Annexin(+)/CD4(+)/PI(-)	3.16 (4.48)	2.33 (7.64)	0.944
Annexin(+)/CD8(+)/PI(-)	3.74 (8,71)	6.47 (15.71)	0.269
Annexin(+)/PI(-) of isolated monocytes	20.62 (28.11)	12.19 (22.98)	0.007

*Natural killer cells were defined as CD3(-)/CD(16+56)(+). Values are expressed as median (IQR) absolute numbers for CD3(+)/CD4(+), CD3(+)/CD8(+), CD3(+)/CD(16+56)(+), natural killer and CD19(+) cells and as median (interquartile range) percentages for Annexin(+)/CD4(+)/PI(-), Annexin(+)/CD8(+)/PI(-) and Annexin(+)/PI(-) of isolated monocytes. VAP = ventilator-associated pneumonia.

**Figure 1:**
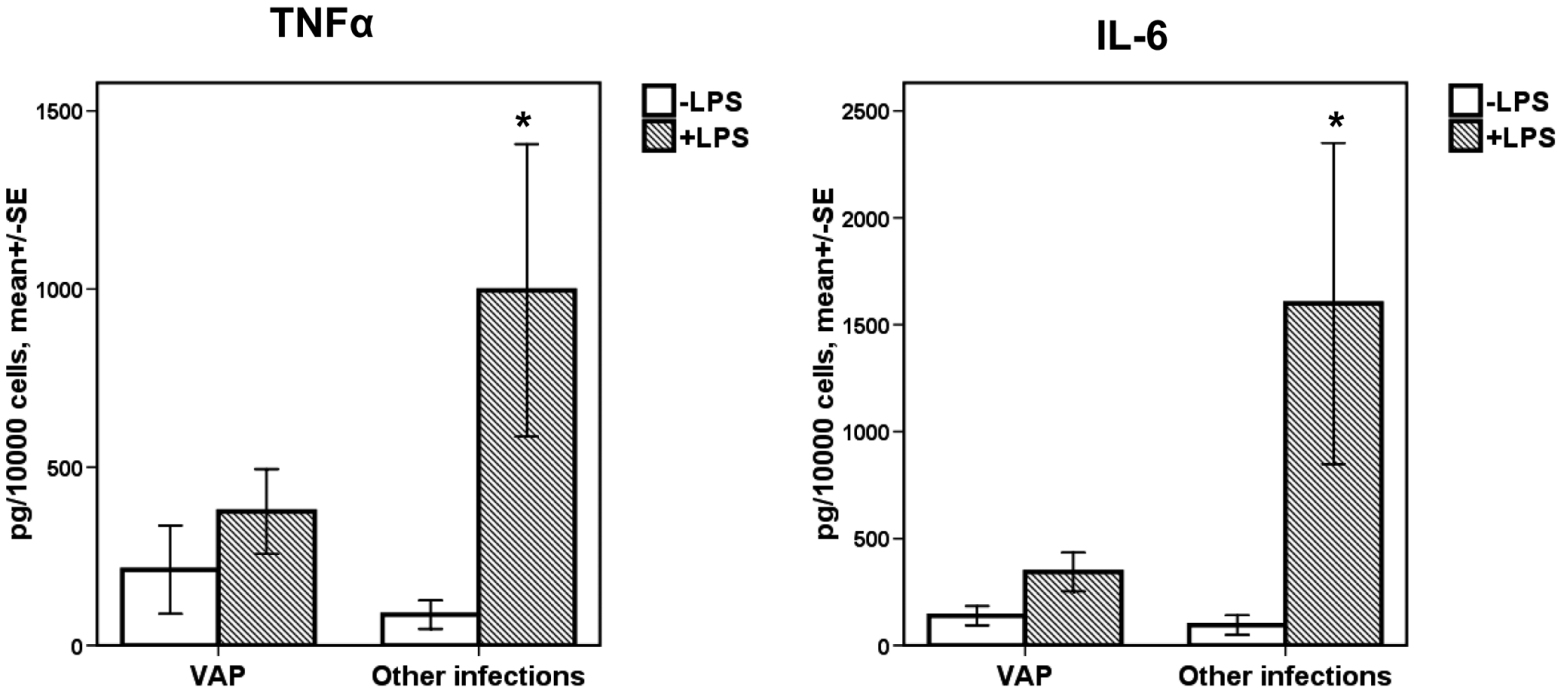
**TNFα and IL-6 production from the supernatants of monocytes**. Concentrations of TNFα and IL-6 of supernatants of monocytes of patients with sepsis due to ventilator-associated pneumonia (VAP) and patients with sepsis caused by other nosocomial infections. The asterisk denotes significant difference between the two groups of patients. (*P *= 0.008 for TNFα; *P *= 0.003 for IL-6). LPS = lipopolysaccharide; SE = standard error.

Cytokine release by monocytes upon stimulation with LPS is shown in Figure 1. Release of both TNFα and IL-6 from monocytes was lower in patients with VAP-related sepsis than with sepsis related to other types of infection.

**Figure 2:**
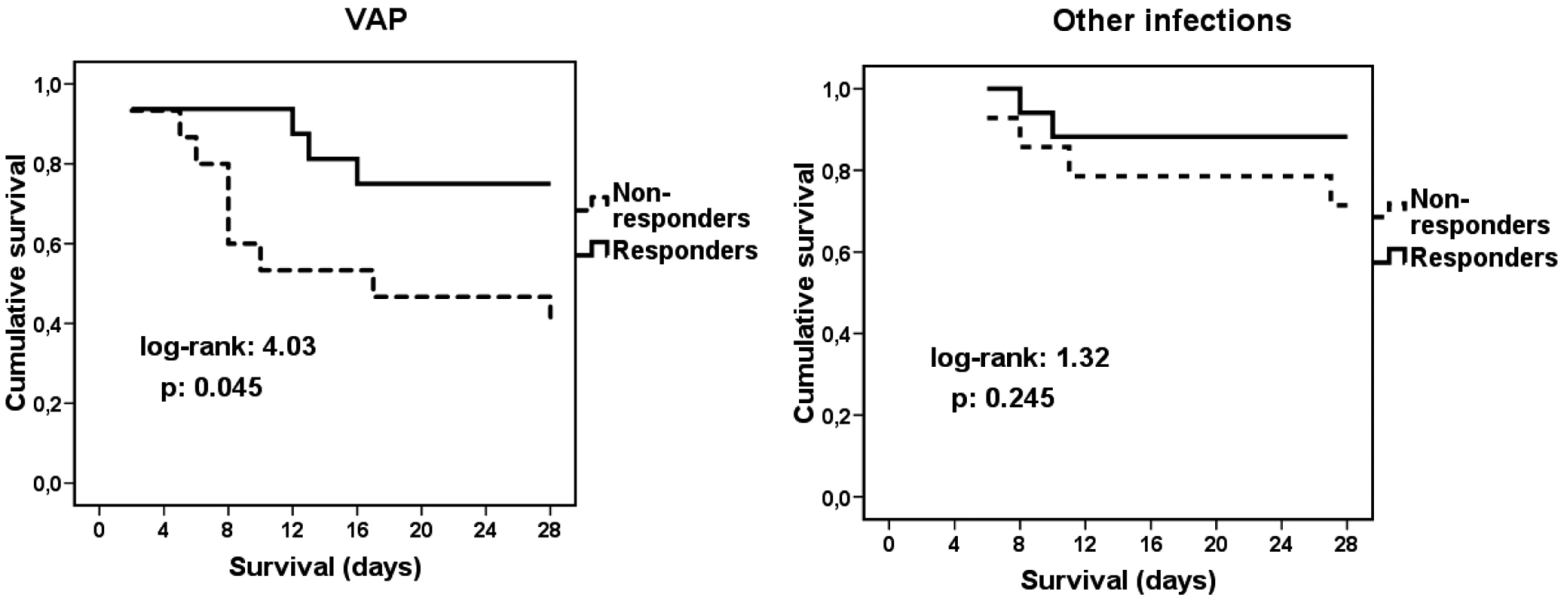
**Comparison of survival of septic patients**. Comparison of survival of septic patients due to ventilator-associated pneumonia (VAP) and patients with sepsis caused by other infections depending on the presence or absence of response of their monocytes to stimulation with lipopolysaccharide.

Kaplan-Meier analysis of survival of patients subgrouped into responders and non-responders after stimulation with LPS revealed that a positive response after stimulation was a detrimental factor affecting survival among patients with sepsis caused by VAP but not in sepsis caused by other infections. More precisely, among patients with VAP-related sepsis, 28-day mortality of responders was 25% compared with 60% of non-responders (*P *= 0.045, Figure 2). Among those with other infections, 28-day mortality of responders was 11.76% and of non-responders 28.57% (*P *= 0.245, Figure 2).

To exclude the possibility that results may be related to the process of mechanical ventilation, patients with non-VAP related-sepsis were further divided in to two subgroups, those being intubated and those not being intubated. No difference in the percentage of CD3(+)/CD4(+) lymphocytes and in the apoptosis of monocytes was observed between the two subgroups. More precisely, median expression of CD3/CD4 on lymphocytes was 49.60% and 54.66%, respectively (*P *= 0.654) and median apoptosis of monocytes was 8.29% and 15.15%, respectively (*P *= 0.329).

**Figure 3:**
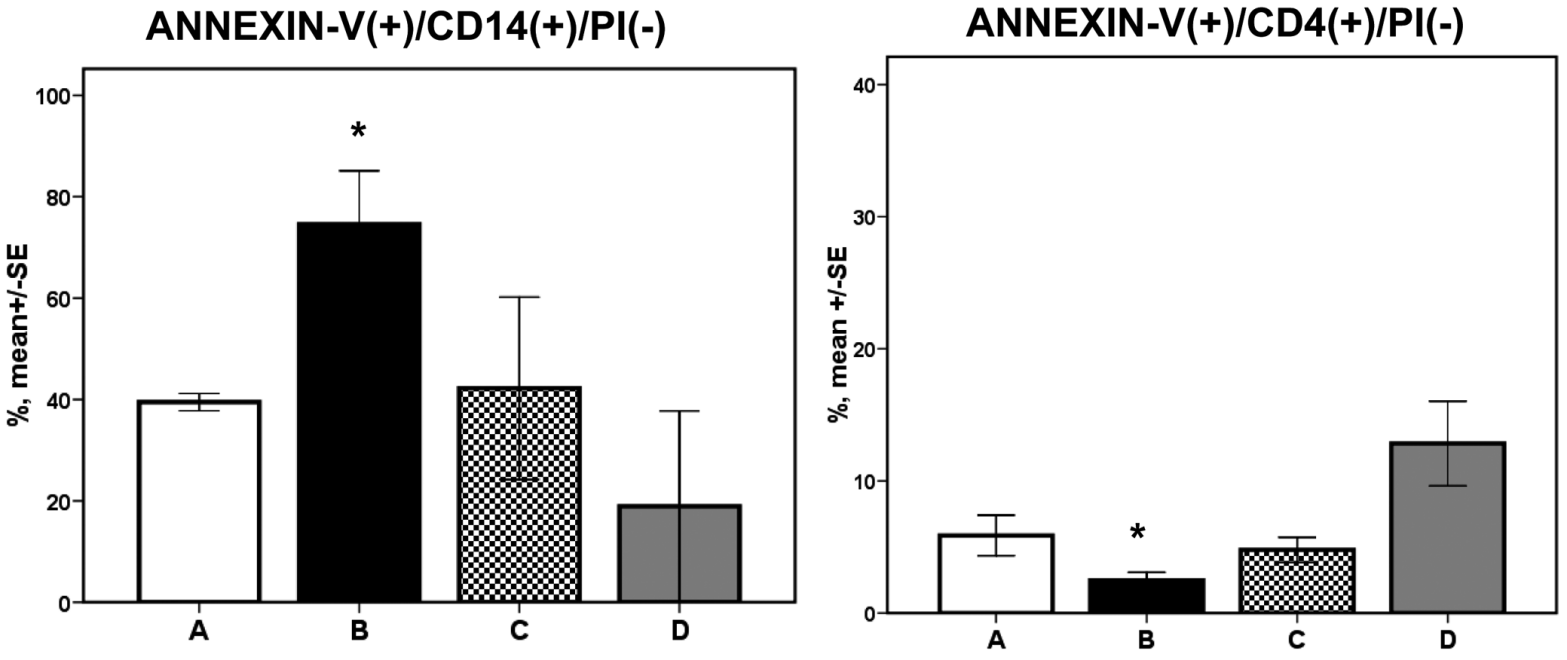
**Apoptosis of CD14-monocytes and of CD4-lymphocytes of healthy volunteers**. Induction of apoptosis of CD14-monocytes and inhibition of apoptosis of CD4-lymphocytes of healthy volunteers according to four different patterns of stimulation by isolates of *Acinetobacter baumannii *and of *Pseudomonas aeruginosa*. A = un-stimulated controls; B = three-step stimulation mimicking pathogenesis of ventilator-associated pneumonia (VAP); C = abrupt stimulation with pathogens of VAP; and D = abrupt stimulation mimicking pathogenesis of bacteremia. Asterisks denote significant difference between patterns B and D and between patterns B and A. SE = standard error.

The rate of apoptosis of lymphocytes and of monocytes for each pattern of stimulation is shown in Figure 3. Stimulation according to pattern B mimicking pathogenesis of VAP was accompanied by inhibition of apoptosis of CD4-lymphocytes and by induction of apoptosis of CD14-monocytes compared with both patterns A and D.

## Discussion

Sepsis is accompanied by dysregulated immune response. Among patients, those with VAP are considered more compromised than others because of the iatrogenic intervention in mechanical lung defenses due to endotracheal intubation [19,20]. A recent publication by our group showed that apoptosis of monocytes in patients with VAP may play a considerable role in the final outcome of the patient [8]. However, the point of discussion is whether this innate immune response is a unique characteristic of sepsis related to VAP or even of sepsis not related to VAP. The present study investigated the alterations of innate and of adaptive immune responses in patients with sepsis due to VAP in comparison to septic patients with other infections. Every attempt was made to match both groups of patients according to age, sex, disease severity and causative pathogens. The latter were Gram-negative species. It has to be emphasized that in the Greek setting, VAP is mainly caused by Gram-negative pathogens [21].

Flow cytometry analysis revealed two major differences between sepsis due to VAP and sepsis caused by other infections. The first difference is the decrease of CD3(+)/CD4(+) lymphocytes in VAP. Depletion of T-helper lymphocytes in sepsis has already been described and attributed to accelerated apoptosis [22]. In the present study, no difference in the apoptotic rate of T-helper lymphocytes between the two groups of patients was shown.

The second major finding is a considerable increase of apoptosis of monocytes in patients with VAP. As a consequence of that phenomenon, immunoparalysis of monocytes, which occurs normally in sepsis [23,24], is pronounced in VAP compared with other infections. Immunoparalysis was stated by the inability of monocytes to produce sufficient amounts of TNFα and IL-6 after stimulation with LPS (Figure 1). Among patients with VAP, those with monocytes responding to LPS stimulation presented a survival benefit compared with non-responders. That was not the scenario for sepsis caused by other types of infection. Although it was obvious that VAP was a situation of profound immunoparalysis, survival was prolonged among those patients with adequate monocyte function (Figure 2).

A question emerging from these results was whether immunoparalysis observed among patients with VAP was a result of their baseline characteristics. The two groups of patients did not differ in sex, age, disease severity or co-morbidities. The use of corticosteroids for the treatment of the septic syndrome was also similar between VAP and non-VAP septic patients. The presence of prior bacterial infections was rare in both groups. The possibility that mechanical ventilation could have acted as a confounding factor was excluded, because no difference was observed when the percentages of T-helper lymphocytes and the apoptosis of monocytes between intubated and non-intubated non-VAP patients were compared. *P. aeruginosa *and *A. baumannii *were more frequently responsible for VAP than for other infections. This was expected because these two microorganisms constitute the two major pathogens of nosocomial pneumonia in Greece [25].

*In vitro *findings support the hypothesis that one major cause of immune alterations in patients with sepsis is the type of contact of immune cells with the pathogens. More precisely, in patients with VAP the immune system is gradually exposed to the pathogen. The latter is entering the airways through aspiration of the oropharyngeal flora and then steadily increases to an amount able to induce VAP. As a consequence, the immune system is gradually exposed to sequentially increased bacterial inocula, which leads to decreased apoptosis of CD4-lymphocytes and to increased apoptosis of CD14-monocytes (Figure 3, pattern B). When VAP evolves abruptly, similar alterations are not seen (Figure 3, pattern C). This is also the case with bacteremia (Figure 3, pattern D).

The *in vitro *experiment was based on the assumption that VAP supervenes as a result of gradual and continuous exposure of the innate immune system to the pathogen while non-VAP sepsis is the result of an abrupt stimulation of the innate immune system. The response of PBMCs of healthy volunteers may differ from those of PBMCs of septic patients. A number of factors participate to the interactions between bacteria and the immune system, such as virulence genes or pattern recognition receptors, whose role was not studied in our setting. Further investigation is mandatory in order to clarify our hypothesis about the pathogenesis of VAP.

## Conclusion

The presented findings reveal that innate and adaptive immune responses differ considerably between sepsis due to VAP and sepsis due to other types of nosocomial infection. VAP is characterized by substantial decrease of CD4-lymphocytes and immunoparalysis of monocytes in contrast to other infections. The mechanism of bacterial pathogenesis of VAP may help explain these differences. The latter could constitute a novel therapeutic target for the management of the septic patient with VAP.

## Key messages

• Sepsis due to VAP is characterized by decrease of CD3/CD4(+) lymphocytes and immunoparalysis of monocytes compared with sepsis caused by other nosocomial infections.

• The mechanism of bacterial pathogenesis of VAP seems to play a crucial role in the explanation of these differences.

## Abbreviations

CAP: community-acquired pneumonia; CPIS: Clinical Pulmonary Infection Score; EDTA: ethylenediamine tetraacetic acid; ELISA: enzyme-linked immunosorbent assay; FBS: fetal bovine serum; FiO2: fraction of inspired oxygen; FITC: fluorescein isothiocyanate; HAP: hospital acquired pneumonia; ICU: intensive care unit; IL-6: interleukin-6; LPS: lipopolysaccharide; PBMCs: peripheral blood mononuclear cells; PBS: phosphate-buffered saline; pCO2: partial pressure of carbon dioxide; PE: phycoerythrin; PI: propidium iodine; pO2: partial pressure of oxygen; TBS: tracheobronchial secretions; TNFα: tumour necrosis factor alpha; VAP: ventilator-associated pneumonia; WBC: white blood cells.

## Competing interests

The authors declare that they have no competing interests.

## Authors' contributions

AP participated in the follow-up of patients, performed the *in vitro *experiments and the estimation of TNFα and IL-6, participated in the immunophenotypic analysis, analysed the data and wrote the manuscript. IT participated in the enrolment and follow-up of patients. AK and VK participated in the immunophenotypic analysis. HG and AA drafted the manuscript. EJG-B participated in the study design and the analysis of data and drafted the manuscript.
